# Real-time holographic camera for obtaining real 3D scene hologram

**DOI:** 10.1038/s41377-024-01730-9

**Published:** 2025-02-08

**Authors:** Zhao-Song Li, Chao Liu, Xiao-Wei Li, Yi Zheng, Qian Huang, Yi-Wei Zheng, Ye-Hao Hou, Chen-Liang Chang, Da-Wei Zhang, Song-Lin Zhuang, Di Wang, Qiong-Hua Wang

**Affiliations:** 1 School of Instrumentation and Optoelectronic Engineering, Beihang University, Beijing, 100191 China; 2 School of Optical-Electrical and Computer Engineering, University of Shanghai for Science and Technology, Shanghai, 200093 China

**Keywords:** Applied optics, Optical techniques, Optical materials and structures

## Abstract

As a frontier technology, holography has important research values in fields such as bio-micrographic imaging, light field modulation and data storage. However, the real-time acquisition of 3D scenes and high-fidelity reconstruction technology has not yet made a breakthrough, which has seriously hindered the development of holography. Here, a novel holographic camera is proposed to solve the above inherent problems completely. The proposed holographic camera consists of the acquisition end and the calculation end. At the acquisition end of the holographic camera, specially configured liquid materials and liquid lens structure based on voice-coil motor-driving are used to produce the liquid camera, so that the liquid camera can quickly capture the focus stack of the real 3D scene within 15 ms. At the calculation end, a new structured focus stack network (FS-Net) is designed for hologram calculation. After training the FS-Net with the focus stack renderer and learnable Zernike phase, it enables hologram calculation within 13 ms. As the first device to achieve real-time incoherent acquisition and high-fidelity holographic reconstruction of a real 3D scene, our proposed holographic camera breaks technical bottlenecks of difficulty in acquiring the real 3D scene, low quality of the holographic reconstructed image, and incorrect defocus blur. The experimental results demonstrate the effectiveness of our holographic camera in the acquisition of focal plane information and hologram calculation of the real 3D scene. The proposed holographic camera opens up a new way for the application of holography in fields such as 3D display, light field modulation, and 3D measurement.

## Abstract (web summary)

A real-time holographic camera for acquiring high-fidelity holograms of real 3D scenes based on a elastic membrane liquid camera and a focal stack network is proposed.

## Graphical abstract

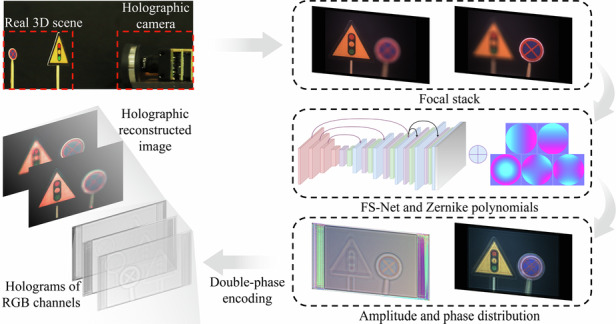

## Introduction

Holography can completely record and recover the wavefront information of a 3D object, which has important research values in bio-micrographic imaging^1–3^, light field modulation^4–7^, data storage^8–10^, encryption^11–13^ and so on. In recent years, in the field of holographic 3D display, researchers have carried out many kinds of frontier research around the fast calculation of holograms^14–16^, large-viewing angle holographic 3D display^17–20^, and low-noise holographic reconstruction^21–23^. However, most of these works take the virtual 3D object instead of the real 3D scene as the recorded object^24,25^. In addition, due to the time-consuming acquisition of the real 3D scene and the use of strong coherent light in the holographic reconstruction, there are still two major bottlenecks in the holographic 3D display that need to be solved: 1) Real-time generation of holograms of the real 3D scene is difficult to achieve^26,27^. 2) The holographic reconstructed image has severe speckle noise and does not match the defocus blur of the real 3D scene^28–30^.

Based on the method of incoherent light recording and optical holographic reconstruction, the holographic camera can achieve the recording and reconstruction of a real 3D scene^31–34^. However, since capturing the real 3D scene and encoding the hologram tend to take a lot of time, the scene capturing speed and hologram calculating speed are not yet able to reach real-time^35–37^. The electrowetting liquid lens is shown to improve the recording speed of the real 3D scene^38,39^. However, the zoom switch time of the electrowetting liquid lens is ~100 ms^31,40^, thus it cannot meet the real-time acquisition requirements of real 3D scenes.

According to the different data representations of the 3D object, the calculations of holograms are mainly classified into point-based methods^41,42^, polygon-based methods^43,44^, and layer-based methods^45,46^. These methods often involve a diffraction calculation process, which leads to a significant decrease in the calculation speed of holograms as the amount of data increases. In recent years, researchers have proposed data-driven and model-driven hologram-based calculation methods using deep neural networks, which effectively improve the quality of the holographic 3D display^47–50^. However, since the inputs to the network are usually the all-in-focus image and depth map of the 3D scene^51–53^, it is not possible to supervise the training of the defocus blur information of the 3D scene, which leads to the mismatch of the defocus blur between the holographic reconstructed image and the real 3D scene. Nowadays, there is an urgent need to develop a holographic 3D display equipment with both recording speed and calculation speed to achieve real-time capturing and high-fidelity holographic reconstruction of the real 3D scene.

**Fig. 1:**
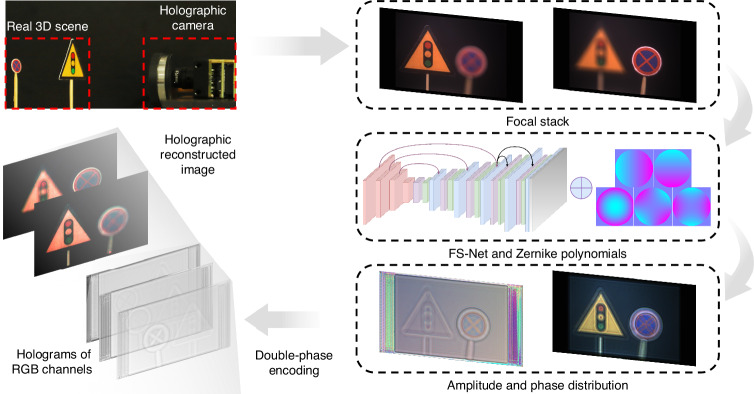
Concept of the proposed holographic camera

In this paper, a holographic camera is proposed for real-time acquisition and hologram generation of the real 3D scene, which consists of two parts: acquisition end and calculation end, as shown in Fig. 1. At the acquisition end, a liquid camera with an independently developed elastic membrane liquid lens actuated by a voice coil motor is used to rapidly acquire multi-focal plane information of the real 3D scene. Benefit by high refractive index filled liquid material and the special elastic membrane surface-type control component, the liquid lens can reach fast zoom function within 15 ms. Meanwhile, the high-precision current driver improves the precision of focal power control, and the liquid camera can accurately acquire the multi-focal plane information of the real 3D scene. At the calculation end, a fast hologram generation method based on the focal stack network (FS-Net) is designed. The focal stack of the real 3D scene captured by the liquid camera is input into the FS-Net, and after complex amplitude distribution calculation and double-phase coding, the holograms of red, green and blue (R, G, B) channels of the real 3D scene are output. In the FS-Net, the up-sampling block, down-sampling block, and skip connection, all based on the pixel shuffle and pixel unshuffle, are designed to realize the real-time calculation of the holograms. The time from scene capture to display is only 28 ms. In addition, a focus stack renderer is introduced during the training process of the FS-Net and the aberration compensation method based on Zernike polynomials is used. Experiments show that the peak signal-to-noise ratio (PSNR) of the reconstructed image can reach 40 dB. The proposed holographic camera is the first camera to achieve real-time capture and display of a real 3D scene, addressing the issue that the blur of the holographic reconstructed image does not match the blur of the real 3D scene. The proposed holographic camera has significant application prospects in the fields including 3D display, 3D measurement and so on.

## Structure and principle

The proposed holographic camera consists of two parts: acquisition end and calculation end. In the acquisition end, a liquid camera based on an elastic membrane liquid lens is proposed to quickly capture the multi-focal plane information of a real 3D scene. In the calculation end, a hologram generation method based on the FS-Net is proposed to calculate the high-fidelity hologram in real-time.

### Design of the liquid camera

The liquid camera consists an elastic membrane liquid lens and a solid lens group, and the elastic membrane liquid lens is the key component enabling the liquid camera zoom function. The focal power *Φ*_C_ of the liquid camera can be expressed as follows:

1 $${{\Phi }}_{C}={{\Phi }}_{L}+{{\Phi }}_{S}-{d}_{LS}\cdot {{\Phi }}_{L}\cdot {{\Phi }}_{S}$$

where *Φ*_L_ and *Φ*_S_ represent the focal power of the liquid lens and the solid lens group, respectively, and *d*_LS_ represents the spacing between the optical principal planes of the liquid lens and the solid lens. When the liquid camera is assembled, the values of *Φ*_S_ and *d*_LS_ will remain constant. When the liquid camera is employed to capture the focus stack of a real 3D scene, the focal power *Φ*_L_ of the liquid lens varies with the drive current. Therefore, the focal power *Φ*_C_ of the liquid camera can be modified by changing the drive current of the liquid lens. Based on this scheme, the liquid camera can accurately and quickly capture the multi-focal plane information of a real 3D scene.

The conventional elastic membrane liquid lenses are usually actuated by hydraulic or pneumatic pressure, and the actuation system is bulky and not easily integrated into optoelectronic systems. To overcome the above limitations, a new type of elastic membrane liquid lens actuated by a voice coil motor is developed. By using special elastic membrane surface control components, the elastic membrane liquid lens can achieve a fast response speed and switchable positive and negative focal lengths. In addition, a high-precision current control driver is developed to ensure that the liquid camera can quickly capture the real 3D scene.

**Fig. 2:**
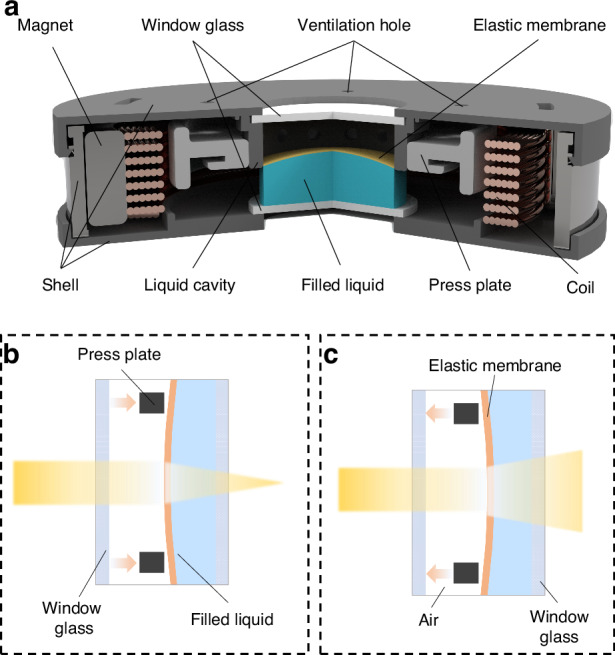
Structure and principle of the elastic membrane liquid lens. **a** Structure of the elastic membrane liquid lens. **b** Convergent state of the elastic membrane liquid lens. **c** Divergent state of the elastic membrane liquid lens

The structure of the elastic membrane liquid lens is shown in Fig. 2a. The elastic membrane liquid lens consists of a shell, two pieces of window glass, a press plate, a liquid cavity, a magnet, a coil, an elastic membrane, filled liquid and several ventilation holes. The coil is in a radial magnetic field constituted by the magnet. The ventilation holes ensure that the air pressure inside the liquid lens is consistent with the outside environment. The press plate is tightly connected to the coil and the elastic membrane. The elastic membrane and the liquid cavity form a confined space. When the drive current is not applied, the elastic membrane forms a natural curved surface at the optical aperture of the lens. When the drive current flows through the coil, according to Ampere’s law, an Ampere force generates, which in turn moves the press plate. The magnitude of the Ampere force *F* can be expressed as follows:

2 $$F={\rm{n}}BIL$$

where *n* is the number of turns of the coil, *B* is the magnetic field strength at the location of the coil, *I* is the magnitude of the drive current, and *L* is the perimeter of the single-turn coil.

The direction of the drive current determines the direction of the Ampere force. When the direction of the Ampere force is to the right, the press plate moves to the right. Since the filled liquid cannot be compressed, the filled liquid gathers toward the center region of the elastic membrane, and the elastic membrane bulges. At this point, the elastic membrane liquid lens forms a convex surface, which converges the light, as shown in Fig. 2b. When the direction of the Ampere force is to the left, the press plate moves to the left. Due to the action of the air in the liquid lens, the filled liquid will be squeezed toward the peripheral region of the elastic membrane, and the elastic membrane dimples. At this moment, the elastic membrane liquid lens forms a concave surface, which disperses the light, as shown in Fig. 2c. Therefore, by controlling the magnitude and direction of the drive current, the magnitude and direction of the Ampere force can be controlled, which changes the focal power of the elastic membrane liquid lens and the type of the elastic membrane surface.

### Principle of the FS-Net-based hologram generation method

**Fig. 3:**
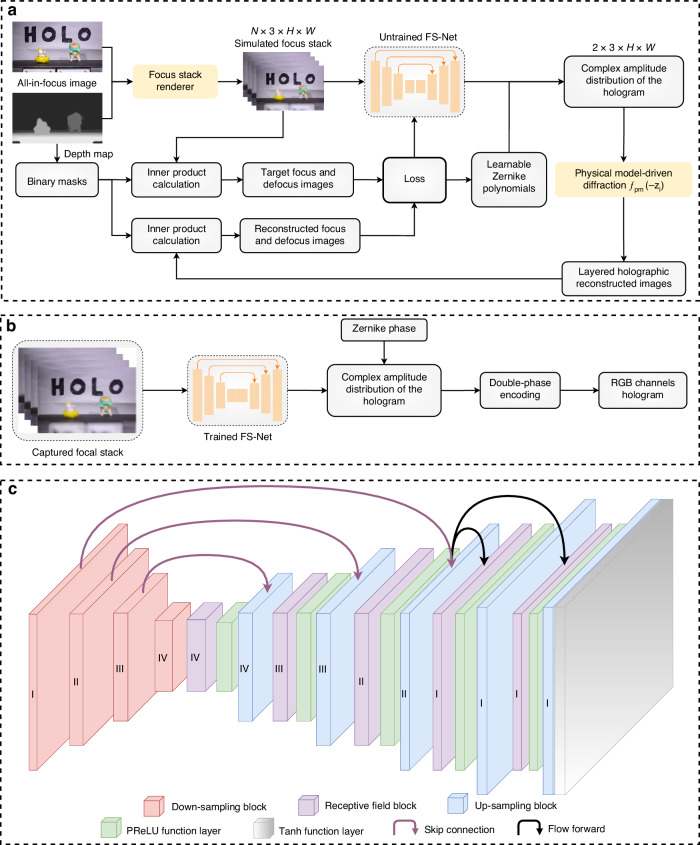
Method of training and generating holograms based on the FS-Net. **a** Training process of the FS-Net. **b** Hologram generation method based on the FS-Net. **c** Detailed structure of the FS-Net

The training flow chart of the proposed FS-Net is shown in Fig. 3a. The innovations of the proposed network are as follows: 1) Different from the traditional deep learning-based hologram calculation methods, the input of the FS-Net is the focus stack of the 3D scene, and the output is the complex amplitude distribution of the hologram of the 3D scene. 2) To simulate the focus stack captured by the liquid camera, a focus stack renderer based on Gaussian blurring and circle of confusion is introduced during the training process of the FS-Net, which is used to realize the fast generation of the focus stack of a 3D scene. The CUDA acceleration technology is used in the focus stack renderer, and the rendering process of the four-layer focus stack can be completed in ~2 s (supplementary information S1). 3) Unlike the common approach of utilizing transposed convolution for tensor calculation, the up-sampling block and skip-connection based on the pixel shuffle are designed in our proposed FS-Net to improve the calculation speed of the model to realize the fast calculation of the hologram. 4) Most of the previous hologram calculation methods do not take into account the correctness of the defocus blur of the holographic reconstructed image. Therefore, the training process of the FS-Net introduces learnable Zernike polynomials for compensating the complex amplitude distribution of the holograms of the 3D scene, which ensures that the holographic reconstructed image matches the defocus blur of the real 3D scene (supplementary information S2).

The training process of the FS-Net consists of five steps. In the first step, the focus stack renderer is utilized to process the all-in-focus image and the depth map of the 3D scene into the focus stack, which has a tensor size of *N*×3×*H*×W (*N* is the number of layers, *H* is the height, and *W* is the width). In the second step, the focus stack is input into the untrained FS-Net. The untrained FS-Net is used to output the complex amplitude distribution of the hologram of the focus stack with a tensor size of 2×3×*H*×*W*. In the third step, the physical diffraction model *f*_pm_ is utilized to back-propagate the complex amplitude distribution with *z*_i_ (*i* = 1, 2, …) distance to obtain the layered holographic reconstructed images. In the fourth step, *N* binary masks are obtained based on the number of layers *N* and the depth map, and then the inner products of the binary masks with the focus stack and the layered holographic reconstructed images are calculated to obtain the target focus and defocus images, and the reconstructed focus and defocus images, respectively. In the fifth step, the loss function between the target focus and defocus images and the reconstructed focus and defocus images is calculated, and the parameters in the untrained FS-Net with learnable Zernike phase are optimized (supplementary information S2). The Zernike phase is helpful for the FS-Net to generate high fidelity holograms. The above steps are repeated and the training of the FS-Net stops when the network iterates for a fixed number of rounds. The hologram generation method based on the trained FS-Net is shown in Fig. 3b. The focus stack captured by the liquid camera is input into the trained FS-Net, which then outputs the complex amplitude distribution of the hologram of the focus stack. After compensating for the complex amplitude distribution using the Zernike phase, the hologram of the focal stack can be obtained by utilizing the double-phase encoding method (supplementary information S3).

As shown in Fig. 3c, the FS-Net is composed of the down-sampling block, the receptive field block, the up-sampling block, the parametric rectified linear unit (PReLU) function layer, and the tangent hyperbolic (Tanh) function layer (supplementary information S4.1). To reduce the parameters in the FS-Net and increase the inference speed of the FS-Net, pixel unshuffle and pixel shuffle are used in the down-sampling block and up-sampling block of the FS-Net, respectively. The receptive field block has a large receptive field, which allows the FS-Net to preserve the image details well. The pixel shuffle-based up-sampling and down-sampling blocks not only preserve the image details, but also can change the image resolution by shuffling pixel information.

The physical model used in the training process of the FS-Net is the band-limited angular spectrum diffraction propagation model (supplementary information S4.2). In addition, to allow the FS-Net to learn the focus information and the defocus information of the 3D scene, the focus loss and the defocus loss are calculated in the loss function of the FS-Net (supplementary information S4.3). The focus loss is used in the supervised training of the target all-in-focus image and the reconstructed all-in-focus image. The total loss is composed of the mean square error (MSE) loss function, the mean absolute error (MAE) loss function, the perceptual (PE) loss function, the multiscale structural similarity (MS-SSIM) loss function and the total variance loss function. The PE loss function ensures that the reconstructed image has realistic texture. The MSE loss function, the MAE loss function, and the MS-SSIM loss function are used in the calculation of the defocus loss for supervised training of the target defocus image and reconstructed defocus image. By reasonably assigning the weights of the focus loss and the defocus loss, effective training of the high-quality hologram of the focus stack can be realized.

## Results

### Fabrication of the liquid camera

**Fig. 4:**
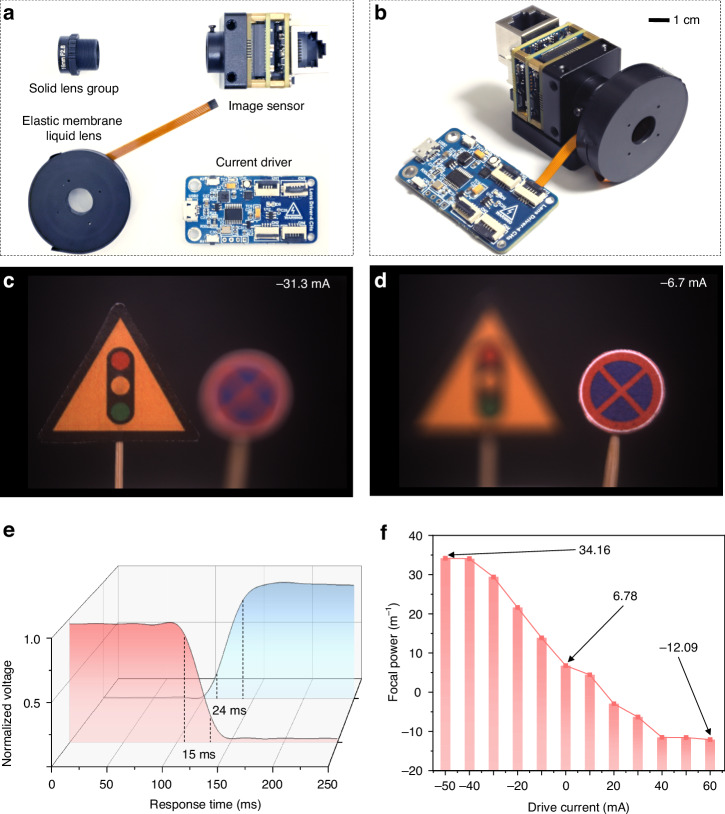
Experimental test of the liquid camera. **a** Components of the liquid camera. **b** Prototype of the liquid camera (scale bar: 1 cm). **c**, **d** Multi-focal information of a real 3D scene captured by the liquid camera. **e** Relationship between the falling and rising response time of the liquid camera and the normalized voltage. **f** Relationship between the focal power of the liquid camera and the drive current

The proposed liquid camera consists of an elastic membrane liquid lens, a solid lens group, an image sensor, and a high-precision current driver, as shown in Fig. 4a and b. The solid lens group is the M12-HF12 manufactured by Shenzhen Jinghang Technology Co. Ltd, with an *F* number of 2.8, a focal length of 16 mm, a thickness of ~19 mm. The image sensor is a Sony IMX178-type photoreceptor chip. The sensor size is 1/1.8 inch, and the pixel size is 2.4 μm. The current driver is developed based on the STM32G070 to provide sufficient drive current for the elastic membrane liquid lens with a current regulation step of ~1 mA. The elastic membrane liquid lens has an optical aperture of 10 mm and a thickness of ~12 mm. The elastic membrane liquid lens can be directly fixed to the solid lens, which is tightly assembled and easy to install.

When the drive currents are -31.3 mA and -6.7 mA, the focal planes of ‘traffic light’ sign and ‘no parking’ sign can be captured by the liquid camera, as shown in Fig. 4c and d, respectively. The distance between the ‘traffic light’ sign and the ‘no parking’ sign is 5 cm. The response time of the liquid camera directly determines the acquisition speed for the 3D scene. The relationship between the response time of the liquid camera and the normalized voltage is shown in Fig. 4e. The falling time and the rising time of the liquid camera is tested to be 15 ms and 24 ms respectively, which endows the holographic camera with the ability to acquire the real 3D scene in real-time (Video-1). The testing method for the response time is described in supplementary information S5.1. When the drive current changes, the focal length of the elastic membrane liquid lens changes, and consequently, the focal power of the liquid camera also changes (supplementary information S5.2). As shown in Fig. 4f, when the drive current changes from -50 mA to 60 mA, the focal power of the liquid camera changes from 34.16 m^-1^ to -12.09 m^-1^, showing a nearly linear negative correlation trend. When capturing a real 3D scene, the depth interval between different focal planes is determined by the adjustment step of the drive current. When the drive current changes by 1 mA, the focusing position of the liquid camera moves ~2 mm. In the experiment of capturing a real 3D scene, the positive focal power part of the liquid camera is mainly relied on.

### Simulation and optical reconstruction experiments

**Fig. 5:**
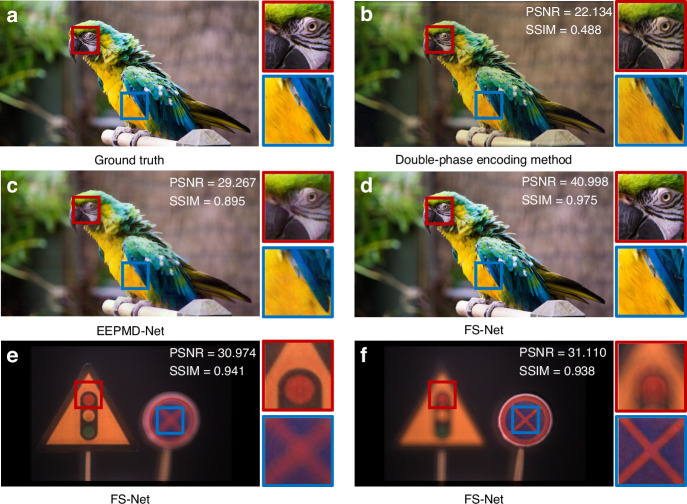
Simulation comparison of different methods. **a** Ground truth of the ‘parrot’. **b-d** Simulation results of the ‘parrot’ by using the double-phase encoding method, EEPMD-Net and FS-Net, respectively. **e**, **f** Simulation results when the ‘traffic light’ sign and the ‘no parking’ sign are focused separately by using the FS-Net

To verify that the proposed FS-Net can better retain the texture and detail information of the input scene, the double-phase encoding method and the end-to-end physical model-driven network (EEPMD-Net)^31^ are used for comparison. The ground truth of the ‘parrot’ is shown in Fig. 5a, and its resolution is 990 × 1760. Figure 5b-d are the simulation results of the ‘parrot’ obtained by using the double-phase encoding method, EEPMD-Net and the proposed FS-Net, respectively. In the simulation experiments, the recording distance for the ‘parrot’ is set to be 5 mm and the wavelengths of the recorded light are 638 nm, 520 nm, and 450 nm, respectively. It can be noticed that the color of the ‘parrot’ obtained by using the double-phase encoding method are distorted, and at the same time, ringing appears in the holographic reconstructed image. The texture of the ‘parrot’ obtained by using the EEPMD-Net is blurred, obviously different from the ground truth. The texture and detail information of the ‘parrot’ obtained by using the FS-Net is fully preserved. The PSNR of the holographic reconstructed image is calculated to be 40.998 dB and the structural similarity (SSIM) is 0.975 (supplementary information S6.1), which prove that the excellent performance of the proposed FS-Net. In addition, no Zernike phase is introduced during the calculation of the hologram of the ‘parrot’. Meanwhile, the time to calculate the holograms of RGB channels of the ‘parrot’ using the FS-Net is ~12 ms, which demonstrates that the proposed FS-Net has the capability for real-time calculation.

When calculating the hologram of the focus stack using the FS-Net, the recording distances for the ‘traffic light’ sign and the ‘no parking’ sign are set to be 5 cm and 10 cm, respectively. The interval between the holographic reconstructed images is 5 cm, which is the same as the actual interval of the recorded 3D scene. The 5 orders Zernike phase is introduced to realize the compensation of the complex amplitude distribution. The calculation time of the holograms of RGB channels of the focus stack using the FS-Net is ~13 ms. The simulation results of the reconstructed focus stack are shown in Fig. 5e and f. When the ‘traffic light’ sign is in focus, there are no obvious diffraction fringes in the defocus ‘no parking’ sign and vice versa. This verifies that the proposed FS-Net can restore the defocus blur of a real 3D scene effectively. It is worth mentioning that the trained FS-Net has high generalization ability. However, the number of layers of the focus stack affects the PSNR and SSIM of the holographic reconstructed image. When the number of layers of the focus stack changes, the complex amplitude distribution of the focus stack also changes, which finally affects the PSNR and SSIM.

**Fig. 6:**
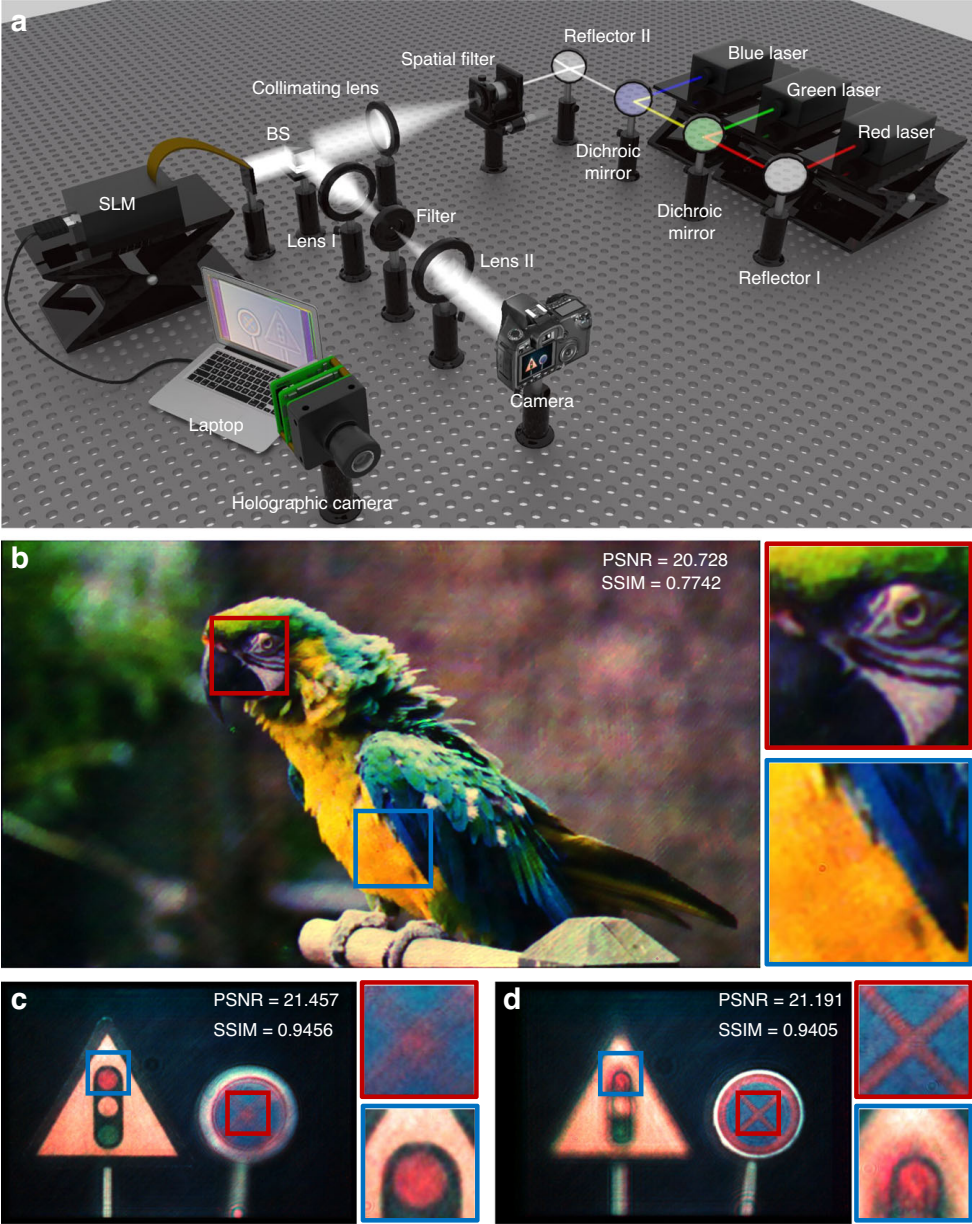
Optical experimental system and reconstruction results. **a** Schematic diagram of the holographic reconstruction system. **b** Color holographic reconstructed image of the ‘parrot’. **c**, **d** Color holographic reconstructed images of the ‘traffic light’ sign and the ‘no parking’ sign when they are focused separately

To verify the advantages of the proposed holographic camera, the holographic reconstruction system of the real 3D scene is built, as shown in Fig. 6a. The holographic reconstruction system consists of the RGB lasers, two dichroic mirrors, two reflectors (including reflector I and reflector II), a spatial filter, a collimating lens, a beam splitter (BS), a spatial light modulator (SLM), a 4 *f* system (including lens I, filter, and lens II), a holographic camera, and a laptop. The wavelengths of the RGB lasers are 638 nm, 532 nm, and 473 nm, respectively. The SLM manufactured by Xi’an CAS Microstar Technology Co. Ltd has a resolution of 1920×1080 and a pixel pitch of 6.4 μm. The Canon EOS 77D is used to capture the holographic reconstructed images. The light emitted from the RGB lasers is combined into a uniform parallel white light after passing through the spatial filter and the collimating lens. Then, the white light passes through the BS and illuminates the SLM. The color holographic reconstructed images modulated by the SLM are filtered by the 4 *f* system and captured by the camera. The optical reconstruction results are shown in Fig. 6b-d.

The color holographic reconstructed image of the ‘parrot’ based on the FS-Net is shown in Fig. 6b. The PSNR of the holographic reconstructed image is 20.728 dB, and the SSIM is 0.7742. By comparing the ground truth and the holographic reconstructed image of the ‘parrot’, it can be found that the profile, fur color, and texture of the ‘parrot’ is effectively preserved, and there is no ringing interference in the holographic reconstructed image. The color holographic reconstructed images of the focus stack captured by the holographic camera is shown in Fig. 6c and d. When the ‘traffic light’ sign is focused, it can be noticed that the defocus blur of the ‘no parking’ sign is close to the defocus blur of the focus stack captured by the liquid camera in Fig. 4c. A similar phenomenon occurs when the ‘no parking’ sign is focused. In addition, the color holographic reconstructed images in Fig. 6c and d are compatible with the simulation results in Fig. 5e and f, which validate the feasibility of the proposed FS-Net in optical reconstruction.

## Discussion

The holographic camera is a powerful tool to acquire and generate the hologram of a real 3D scene. In this paper, we propose a novel holographic camera consisting of a liquid camera based on an elastic membrane liquid lens and the FS-Net. The demonstrated liquid camera is capable of real-time acquisition of a real 3D scene. Meanwhile, the FS-Net can realize the real-time and high-quality calculation of the hologram of the real 3D scene based on the focus stack of the real 3D scene captured by the liquid camera. In addition, the focus stack renderer and Zernike phase used in the training process of holograms provide a solution to the problem of inaccurate defocus blur of the holographic reconstructed image. The performance of the proposed holographic camera is compared with other work in Table 1. Compared with the existing literature reports, the proposed holographic camera shows a 400% increase in response speed and a 370% reduction in the calculation time of the hologram, and the simulated holographic reconstructed image achieves a PSNR of more than 40 dB^31,34,49^. In this paper, the holograms are trained for the focus stack with two focal planes. In fact, by modifying the parameters of the focus stack renderer, the generation of the focus stack containing more focal planes can be realized with the known all-in-focus image and depth map of a 3D scene. However, with the increase of the number of layers in the focus stack, the calculation time increases accordingly, and the increase of calculation time is nonlinear. In addition, although the FS-Net proposed in this paper only realizes the training of holograms of the focus stack, it is worth mentioning that the input focus stack contains the depth information of the real 3D scene, that is, the FS-Net can also be used for tasks such as depth estimation and depth-of-field fusion. In future work, investigating calculation methods for higher-resolution holograms and designing better network structures are key to further improve the performance of the holographic camera.

**Table 1:** Comparison of characteristics between the proposed method and other methods

Method	Hologram resolution	Response speed	Calculation time	PSNR (Simulation)
Proposed	1920 × 1080	<25 ms	~13 ms	>40 dB
Wang et al. ^31^.	1920 × 1080	91 ms	~53 ms	~28 dB
Shi et al. ^49^.	1920 × 1080	None	~16 ms	~23 dB
Yu et al. ^34^.	720 × 720	100 ms	48 ms	~18 dB

The response time of the elastic membrane liquid lens is usually affected by many factors, such as the size of the lens aperture, the adjustable range of the focal power and the viscosity of the filled liquid. Theoretically, the liquid lens with a small aperture, small focal power range and low viscosity liquid will have a fast response time^54,55^. The aperture size of the proposed liquid lens can reach 10 mm and the focal power can be adjusted from positive to negative. In our experiments, when the liquid lens is driven, the displacement of the center of the elastic membrane can reach ~4 mm, which will affect the response time. Meanwhile, the filled liquid in the proposed liquid lens is a mixture of propylene glycol and tetrabutylammonium chloride with a viscosity of ~56 mPa·s. The resistance of the liquid material with high viscosity will be greater, which will also affect the response time. It is worth mentioning that the mixture is chosen as the filled liquid because it has a higher refractive index, which allows a wider range of focal power adjustment and can make the liquid lens have relatively high mechanical stability. If the aperture of the liquid lens is reduced, deionized water is selected as the filled liquid, and only the liquid lens is driven to the positive state, the response time will be greatly reduced. However, when the aperture and liquid material change, the focal power may be affected, and we need to make a trade-off.

In this paper, a holographic camera is proposed for the real-time capture of a real 3D scene and the real-time generation of the hologram. At the acquisition end of the holographic camera, the elastic membrane liquid lens with a fast zoom response speed is the core device of the liquid camera, which is capable of acquiring the focus stack of a real 3D scene in real-time with a given current. At the calculation end of the holographic camera, the FS-Net is able to realize the fast calculating of the complex amplitude distribution of the hologram of the focus stack. Meanwhile, benefiting from the focus stack renderer and Zernike phase used in the FS-Net training process, the FS-Net is able to realize a natural defocus blur similar to that of a real 3D scene under coherent light illumination. In general, our proposed holographic camera is able to accomplish the acquisition of the focus stack and the fast calculation of the corresponding hologram within 28 ms. The PSNR of the simulated holographic reconstructed image is up to 40 dB. The proposed holographic camera is expected to be applied in the fields of 3D display, 3D measurement, and optical modulation.

## Materials and methods

### Training details

The FS-Net is built using the Pytorch deep learning framework. Pycharm integrated development tool is the platform for training the FS-Net. By using the torch-summay library of Pytorch, the total parameters of the FS-Net are 2889864, the trainable parameters are 2889864, and the memory occupied by the parameters is 11 MB. The DIV2K HD dataset and the MiDaS depth estimation method are used to generate the RGB images and depth maps input to the focus stack renderer. The DIV2K HD dataset contains a total of 900 images with 2K resolution, where 800 images are used to train the network and 100 images are used to validate the network. During the training of the FS-Net, AdamW optimizer is used. Compared with Adam optimizer, AdamW optimizer incorporates weight decay and has a faster convergence speed. The hyperparameters of AdamW optimizer are default values except for the learning rate, which has an initial value of 0.0009. The number of training epochs for the FS-Net is 50, and the RGB channels are trained individually for each input image. When the batch size of the training is 1, the training time is ~50 minutes per round. The model is trained on a computer running Windows 11 professional operating system with an Intel Core i7-12700KF CPU and an NVIDIA® GeForce RTX 3090 GPU (see supplementary information S6.2 for loss curves).

## Supplementary information

Supplementary information

Video 1. Real 3D scene captured by the liquid camera in real-time

## Data Availability

All data and materials that support the results of this work are available from the corresponding authors upon reasonable request.
