# Supplementary material for: Real-time holographic camera for obtaining real 3D scene hologram
Source: Light Sci Appl. 2025 Feb 8;14:74. doi: 10.1038/s41377-024-01730-9 (PMC11806008; doi:10.1038/s41377-024-01730-9)
Supplement: Supplementary file 1 — Supplementary information [file 41377_2024_1730_MOESM1_ESM.docx]

SUPPLEMENTARY INFORMATION

**Real-time holographic camera for obtaining real 3D scene hologram**

Zhao-Song Li^1, †^, Chao Liu^1, †^, Xiao-Wei Li^1^, Yi Zheng^1^, Qian Huang^1^,

Yi-Wei Zheng^1^, Ye-Hao Hou^1^, Chen-Liang Chang^2^, Da-Wei Zhang^2^,

Song-Lin Zhuang^2^, Di Wang^1, *^, and Qiong-Hua Wang^1, *^

^1^ *School of Instrumentation and Optoelectronic Engineering, Beihang University, Beijing 100191, China.*

^2^ *School of Optical-Electrical and Computer Engineering, University of Shanghai for Science and Technology, Shanghai, 200093, China.*

^†^*These authors contributed equally to this work.*

**Correspondence: D Wang, Email: [diwang18@buaa.edu.cn;](mailto:diwang18@buaa.edu.cn;)*

*QH Wang, E-mail: [qionghua@buaa.edu.cn](mailto:qionghua@buaa.edu.cn)*

11 pages, 9 figures, S1-S6

**S1: Principle of the focal stack renderer**

During the training process of the focus stack network (FS-Net), the focus stack renderer is used to generate the focus stack of the 3D scene. An defocus blur generation technology based on the circle of confusion (COC) and Gaussian blur kernel is used in this renderer^1^. COC is often used to quantify the degree of defocus blur. It is considered that there is a thin lens imaging system as shown in Fig. S1a, where the focal length of the thin lens is *f*, and the aperture of the thin lens is *D*. The optical axis of the thin lens has a point *A* and a point *B* emitting light rays respectively. After passing through the thin lens, point *A* is imaged at *A*’ and point *B* is imaged at *B*’. *A*’ is located in front of the complementary metal-oxide-semiconductor (CMOS) and *B*’ is located exactly on the CMOS.


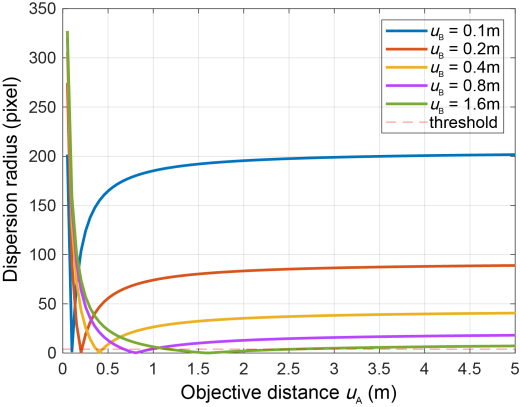

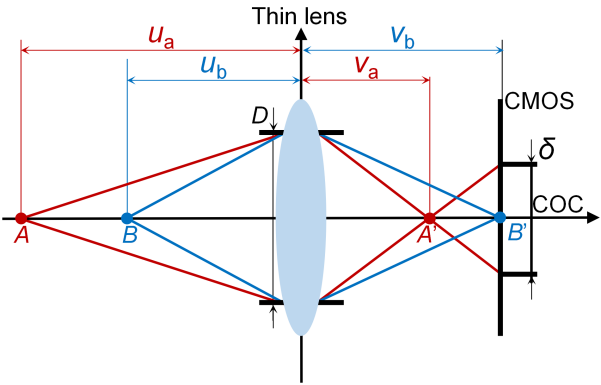


**a**

**b**

**Fig. S1 Physical principles of the focal stack renderer. a** Schematic diagram of thin lens imaging system. **b** Schematic diagram of the variation of the dispersion radius *σ* with respect to the object distance *u*_A_ at different focusing positions *u*_B_.

According to the imaging formula, the relationship between the object distance *u*_A_ at point *A*, the object distance *u*_B_ at point *B*, the image distance *v*_A_ at point *A*, the image distance *v*_B_ at point *B* and the focal length *f* of the thin lens can be expressed as follows:

$\frac{1}{u_{\text{A}}}+\frac{1}{v_{\text{A}}}=\frac{1}{f}$ (S1)

$\frac{1}{u_{\text{B}}}+\frac{1}{v_{\text{B}}}=\frac{1}{f}$ (S2)

According to the similar triangle relationship in Fig. S1a, the mathematical relationship between the aperture *D* of the thin lens and the length *δ* of the COC can be obtained as follows:

$\delta=\frac{f^{2}}{F}\cdot\frac{\left|u_{\text{A}}-u_{\text{B}}\right|}{u_{\text{A}}\text{(}u_{\text{B}}-f\text{)}}$ (S3)

where *F* represents the aperture number, *F*=*f*/*D*, and | | represents the absolute value taken within the symbol. Since COC shows the blurring area of the CMOS, and thus the dispersion radius *σ* can be defined to represent the number of blurring pixels on the CMOS:

$\sigma=\frac{f^{2}}{2p\cdot F}\cdot\frac{\left|u_{\text{A}}-u_{\text{B}}\right|}{u_{\text{A}}\text{(}u_{\text{B}}-f\text{)}}$ (S4)

where *p* represents the pixel pitch of the CMOS. According to Eq. S4, it can be found that when the parameter aperture number *F*, the focal length *f* of the thin lens, the aperture *D* of the thin lens, and the pixel pitch *p* of the CMOS are fixed, the dispersion radius *σ* of the thin lens imaging system is only related to the object distance *u*_A_ and the focusing positions *u*_B_.

The relationship between the dispersion radius *σ* and the object distance *u*_A_ for different focusing positions *u*_B_ is shown in Fig. S1b. As the object distance *u*_A_ changes, the magnitude of the dispersion radius *σ* is different for different focusing positions *u*_B_. When the focusing position *u*_B_ is small, the change of the dispersion radius *σ* is more drastic. As the focusing position *u*_B_ increases, the change of the dispersion radius *σ* tends to level off. Fig. S1b is plotted with an aperture number *F* of 1.5, a focal length *f* of the thin lens of 18 mm, an aperture *D* of the thin lens of 12 mm, and a pixel pitch *p* of the CMOS of 6.4 μm. The threshold value is 1 in Fig. S1b, which means that an object at this distance can be clearly imaged on the CMOS when the dispersion radius *σ*<1. In order to match the virtual rendered focus stack and the actual captured focus stack as well as possible, the parameters in the focus stack renderer need to be set appropriately.

To realize the rendering of the focus stack, it can be considered to convolve the all-in-focus image with a point spread function (PSF). The PSF can be used to describe the propagation process from the point source to the CMOS. To realize the defocus blur, a Gaussian kernel can be selected as the PSF, and the dispersion radius *σ* is just the standard deviation of the Gaussian kernel:

$\text{PSF(}x,y\text{)}=\frac{1}{2\pi\cdot\sigma_{u_{\text{A}}\text{,}u_{\text{B}}}^{2}}\cdot\text{exp(}-\frac{x^{2}+y^{2}}{2\sigma_{u_{\text{A}}\text{,}u_{\text{B}}}^{2}}\text{)}$ (S5)

where (*x*, *y*) represents the spatial coordinates of the all-in-focus image, and the standard deviation of the Gaussian kernel is related to *u*_A_ and *u*_B_. Therefore, the focus stack *J* can be expressed as the convolution of the all-in-focus image *I* with the PSF:

$J\text{=}I*\text{PSF(}x\text{, }y\text{)}$ (S6)

where * represents the convolution operation. By sliding the PSF through the image space, the focus stack *J* can be generated. The depth range of the all-in-focus image is determined by the pixel values of the depth map. The depth segmentation strategy adopted for the depth map in this work is equally spaced segmentation based on the number of layers.

Based on the focus stack renderer described above, the effect of the partial focus stack used in this work to train the FS-Net is shown in Fig. S2. The number of focal planes in Fig. S2 is set to be 4, and the depth of each focal plane is 0.01 m, 0.013 m, 0.02 m, and 0.04 m, respectively. The size of the Gaussian kernel is set to be 21×21. The aperture number *F* of the focus stack renderer is 0.8, the focal length *f* of the thin lens is 8 mm, the aperture *D* of the thin lens is 10 mm, and the pixel pitch *p* of the CMOS is 3.5 μm. The rendering time for the 4 focal plane focus stack is ~2 s.


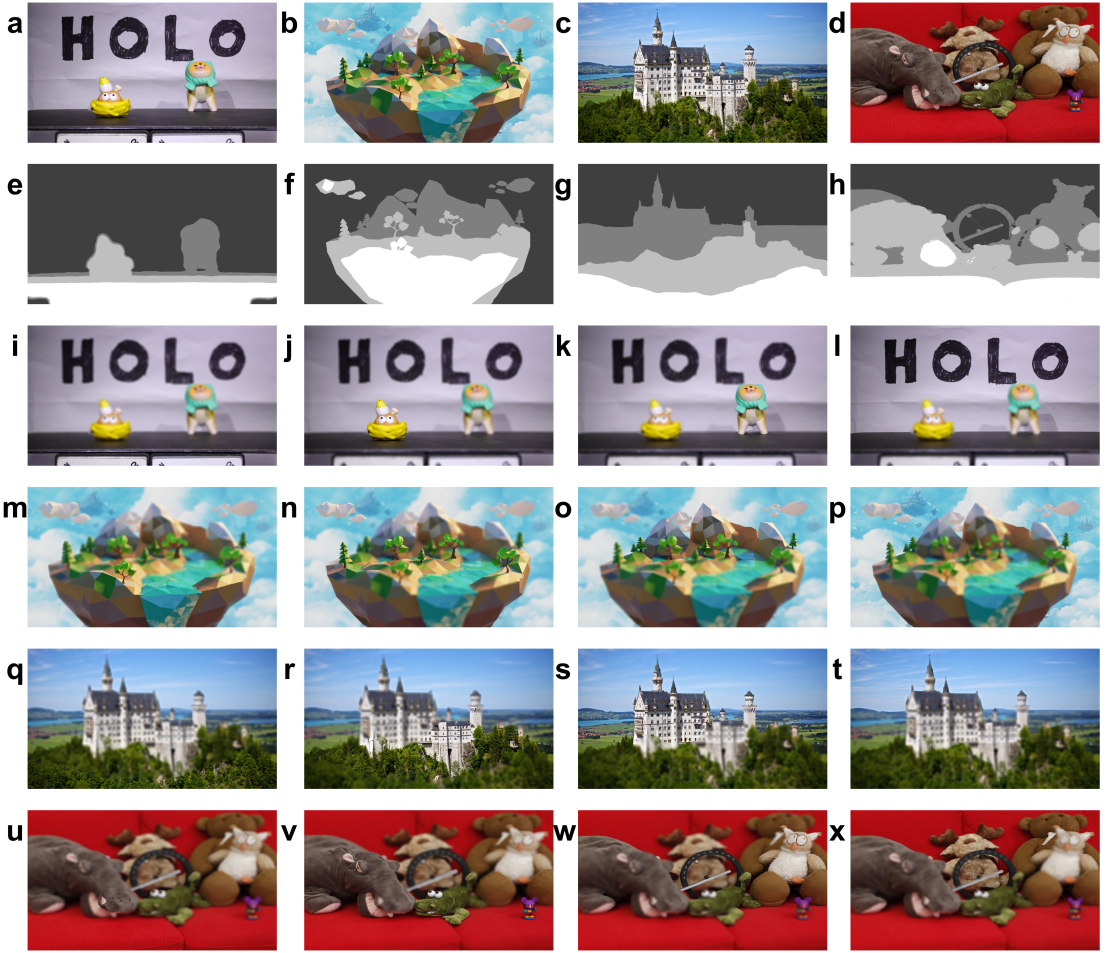


**Fig. S2 Experimental effects based on the focus stack renderer. a-d** All-in-focus image of the 3D scene. **e-h** Depth map of the 3D scene. **i-x** Focus stacks of four different scenes obtained by using the focus stack renderer.

**S2: Principle of the Zernike phase**

Zernike polynomials are a set of functions orthogonal to the unit circle. The aberration of an optical system can be quantified by using Zernike polynomials^2^. Zernike polynomials can decompose the aberration of a wavefront. The commonly used Zernike polynomials have 35 terms of order 7. Among them, order 0 to 2 are low-order aberrations, which can be corrected with a spherical cylinder lens. Order 3 and above are high-order aberrations, which cannot be corrected with a spherical cylinder lens. Since the defocus blur of the holographic reconstructed image is also essentially an aberration, the complex amplitude distribution of the focus stack can be compensated by using 5 terms of Zernike polynomials during the training process of the FS-Net. The expression of the Zernike polynomials is as follows:

$Z_{\text{i}}\text{(}\rho\text{, }\theta\text{)}=\left\{\begin{matrix} Z_{3}\text{(}\rho\text{, }\theta\text{)=}a_{3}\text{(2}\rho^{2}-1\text{)} \\ Z_{4}\text{(}\rho\text{, }\theta\text{)=}a_{4}\text{(}\rho^{2}\text{cos}2\theta\text{)} \\ Z_{5}\text{(}\rho\text{, }\theta\text{)=}a_{5}\text{(}\rho^{2}\text{sin}2\theta\text{) } \\ Z_{6}\text{(}\rho\text{, }\theta\text{)=}a_{6}\text{((3}\rho^{2}-2\text{)}\rho\text{cos}\theta\text{)} \\ Z_{7}\text{(}\rho\text{, }\theta\text{)=}a_{7}\text{((3}\rho^{2}-2\text{)}\rho\text{sin}\theta\text{)} \end{matrix}\right.$ (S7)

where *a*_3_ to a_7_ represent the coefficients of the Zernike polynomials, *ρ* represents the polar radius, and *θ* represents the azimuthal angle. *Z*_3_ controls the defocus of the image, *Z*_4_ and *Z*_5_ control the astigmatism of the image in the vertical and horizontal directions, and *Z*_6_ and *Z*_7_ control the coma of the image in the vertical and horizontal directions. To realize the automatic differentiation calculation of the Zernike polynomials, *a*_3_ to *a*_7_ are set as learnable parameters and *Z*_3_ to *Z*_7_ are non-differentiable Zernike polynomial bases.

In this work, Zernike polynomials are used in training the complex amplitude distribution of the focus stack with two focal planes. After the training, the values of *a*_3_ to *a*_7_ in the Zernike polynomials for the red channel are -0.4370, -0.5275, -0.0062, -0.0006, and 0.0267, respectively. The values of *a*_3_ to *a*_7_ in the Zernike polynomials for the green channel is -0.3849, -0.2520, 0.0022, 0.0006, and 0.0350, respectively. The values of *a*_3_ to *a*_7_ in the Zernike polynomials for the blue channel are -0.0935, -0.1586, 0.0414, -0.0275, and 0.0638, respectively. The Zernike phase distributions of used to compensate for the complex amplitude distribution of the focal stack are shown in Fig. S3.


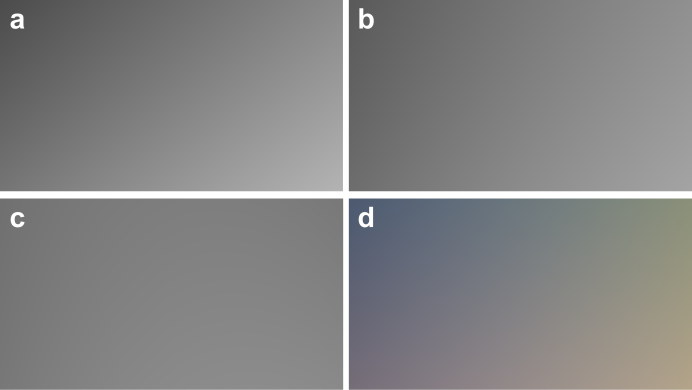


**Fig. S3 Zernike phase distribution obtained from training. a** Zernike phase distribution for the red channel. **b** Zernike phase distribution for the green channel. **c** Zernike phase distribution for the blue channel. **d** Zernike phase distribution for the RGB channels.

After the coordinate transformation, the Zernike phase consisting of the Zernike polynomials in Eq. S7 is multiplied by the complex amplitude distribution *U*_H_ of the uncompensated focal stack, and the complex amplitude distribution *U*_H_’ of the compensated focal stack can be expressed as:

$U_{\text{H}}^{\text{'}}\text{=}U_{\text{H}}\cdot\text{exp(i}\cdot\sum_{j=3}^{7} Z_{\text{j}}\text{(}\rho\text{cos}\theta=x,\rho\text{sin}\theta=y\text{))}$ (S8)

**S3: Principle of the double-phase encoding method**

In this work, the double-phase encoding method is used to encode the complex amplitude distribution of the focus stack generated by the FS-Net into a phase-only hologram^2^. For a given complex amplitude distribution *C*, the expression is as follows:

$C\text{=}A\cdot\text{exp(i}\cdot\alpha\text{)}$ (S9)

where *A* is the amplitude of the complex amplitude distribution *C*, *α* is the phase of the complex amplitude distribution *C*, and *j* represents the imaginary number. The amplitude *A* is normalized to *Ã* and the difference between the phase *α* and the mean value of the phase *ᾱ* is taken. From this, the expressions for the two phase distributions *H*_1_ and *H*_2_ are obtained as follows:

$H_{1}\text{=(}\alpha-\bar{\alpha}\text{)}+\text{cos}^{-1}\text{(}\tilde{A}\text{)}$ (S10)

 (S11)

where cos^-1^ represents the inverse trigonometric function of cos. In order to fuse *H*_1_ and *H*_2_ into the final phase-only hologram, it is necessary to generate the checkerboard patterns *P*_1_(m, n) and *P*_2_(m, n) with the following equations:

 (S12)

 (S13)

where *m* and *n* are the positional indices of the checkerboard pattern. From Eqs. S10-S13, the expression of the phase-only hologram *H* is given as follows:

 (S14)

Some of the holograms generated using the proposed method are shown in Fig. S4. Figs. S4a-d are holograms of the red, green, blue, and color channels for the ‘traffic light’ sign and the ‘no parking’ sign. Figs. S4e-h are holograms of the red, green, blue, and color channels for the ‘parrot’.


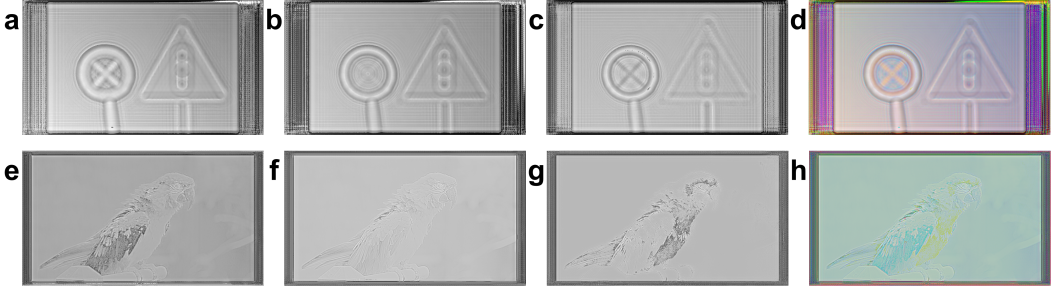


**Fig. S4 Holograms used in the optical experiments. a-d** Holograms of the red, green, blue, and color channels for the ‘traffic light’ sign and ‘no parking’ sign. **e-h** Holograms of the red, green, blue, and color channels for the ‘parrot’.

The focus stack of two focal planes generated using the focus stack renderer and the corresponding simulated holographic reconstructed image are shown in Fig. S5. The number of focal planes of the focal stack renderer is set to be 2, and the depth of each focal plane is 0.05 m and 0.10 m, respectively. The size of the Gaussian kernel is set to be 51×51. The aperture number *F* of the focal stack renderer is 0.8, the focal length *f* of the thin lens is 8 mm, the aperture *D* of the thin lens is 10 mm, and the pixel pitch *p* of the CMOS is 3.5 μm. The reconstructed distances of holographic reconstructed images at the two planes are 0.05 m and 0.10 m, respectively, which are consistent with the depth of the focal plane in the focus stack renderer. It can be found that the defocus blur in the simulated holographic reconstructed image is close to the defocus blur in the focus stack, although there is still a portion of interference fringes appearing in the holographic reconstructed images, which can be solved by further iterative training of the FS-Net.


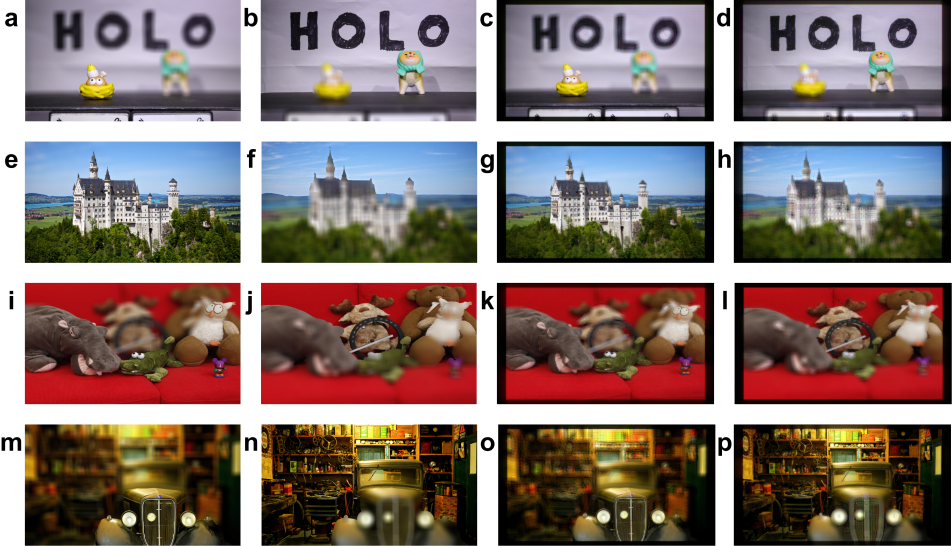


**Fig. S5 Input focus stacks and corresponding simulated holographic reconstructed images. a-b, e-f, i-j, m-n** Focus stacks of two focal planes generated by using the focus stack renderer. **c-d, g-h, k-l, o-p** Simulated holographic reconstructed images based on the FS-Net.

**S4: Details of the training of the FS-Net**

**S4.1: Structure of the FS-Net**

The structure of FS-Net is shown in Fig. S6. The FS-Net consists of three skip connections, four down-sampling blocks, five receptive field blocks, five up-sampling blocks, five parametric rectified linear unit (PReLU) function layers, and a tangent hyperbolic (Tanh) function layer. When the *N* image tensor of the focus stack is input to the down-sampling blocks, the channels of the image tensor are increased and the height and width of the image tensor are halved. For example, as shown in Fig. S6, the shape of the output tensor of down-sampling block I is (*N*, 16, *H*/2, *W*/2), which means that when the image tensor passes through down-sampling block I, the channel of the image tensor is changed from *N* to 16, and the height and width of the image tensor are changed from *H* and *W* to *H*/2 and *W*/2, respectively.


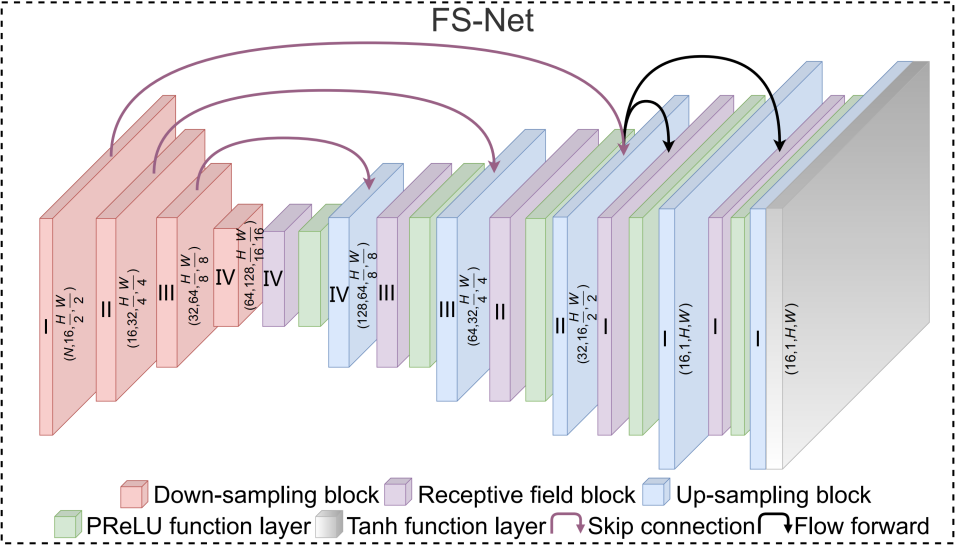


**Fig. S6 Detailed structure of the FS-Net.**

After the four down-sampling blocks, the height and width of the image tensor become 1/16 of the input and the channels become 128. Subsequently, the image tensor enters the receptive field block. In the receptive field block, the convolution kernel with different receptive fields is designed, thus realizing the full extraction of the feature information of the image tensor. The receptive field block does not change the channel, height, and width of the image tensor. After passing through the receptive field block, the image tensor enters the PReLU function layer. The PReLU function layer is used to ensure stable training of the FS-Net. After passing through the PReLU function layer, the image tensor enters the up-sampling block. After passing through the up-sampling block, the channels of the image tensor are reduced and the height and width of the image tensor are doubled. For example, as shown in Fig. S6, the shape of the output tensor of up-sampling block I is (16, 1, *H*, *W*), which means that when the image tensor passes through the up-sampling block I, the channel of the image tensor changes from 16 to 1, and the height and width of the image tensor change from *H*/2 and *W*/2 to *H* and *W*, respectively.

The output of the down-sampling block is added to the output of the up-sampling block passed by the skip connection before proceeding to the next receptive field block. The skip connection is used to pass the shallow information extracted by the network to the deeper layers. For example, a skip connection is used between the output of down-sampling block III and up-sampling block Ⅳ.

After passing through the network structure consisting of the receptive field block, the PReLU function layer, and the up-sampling block three times, the channels of the image tensor become 16, and the height and width become 1/2 of the input. At this point, the image tensor flows forward into two branches. One branch is output after passing through the receptive field block, the PReLU function layer, and the up-sampling block, and the output is the amplitude part of the complex amplitude distribution of the inputting focus stack. The other branch is output after the receptive field block, the PReLU function layer, the up-sampling block, and the Tanh function layer, and the output is the phase part of the complex amplitude distribution of the inputting focal stack.

The location and number of receptive field blocks in the FS-Net are determined after testing. The specific test method is to compare the training loss decline rate of different network structures within the same epochs with the same training parameters and dataset. It can be found that setting the receptive field block before the up-sampling block facilitates the convergence of the FS-Net.

Figs. S7a-c illustrate the structures of the down-sampling block, the up-sampling block, and the receptive field block. Each down-sampling block consists of sequential nets I-III, two pixel unshuffles, and two 1×1 convolution kernels with a step size of 1 (represented as conv2d(1,1) in Fig. S7a). Each up-sampling block consists of sequential nets IV-VI, two pixel shuffles, and two conv2d(1,1). Each receptive field block consists of branches I-IV, two conv2d(1, 1) and a concatenate operation.


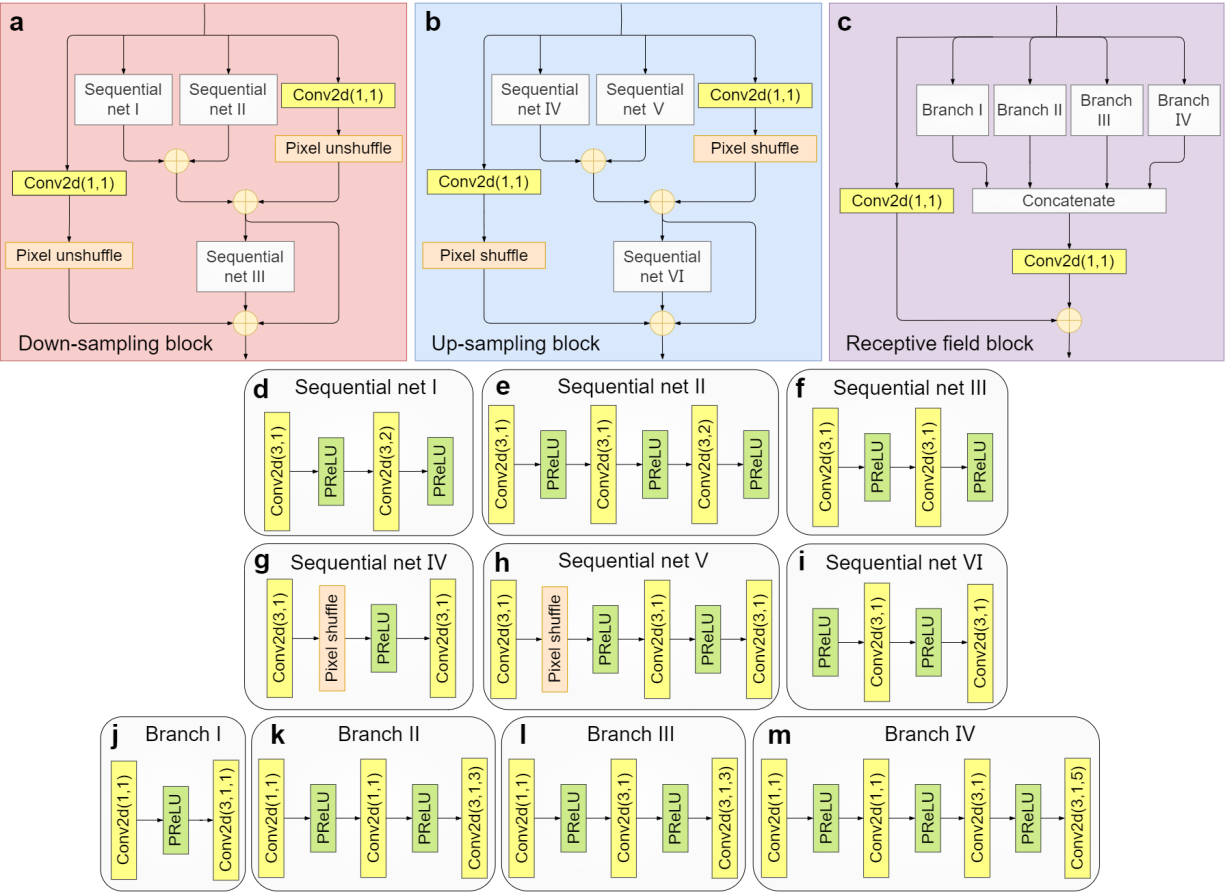


**Fig. S7 Structures of the down-sampling block, up-sampling block, and receptive field block. a** Structure of the down-sampling block. **b** Structure of the up-sampling block. **c** Structure of the receptive field block. **d–i** Structures of sequential nets I–Ⅵ. **j–m** Structures of branches I–IV.

The structures of sequential nets I-VI and branches I-IV are shown in Figs. S7d-m. Since the batch size of the FS-Net is 1, the batch normalization method is not used in the FS-Net. As shown in Figs. S7g-i, instead of using transposed convolution to accomplish up-sampling, pixel shuffle is used in the FS-Net. Since there are no learnable parameters in pixel shuffle, the use of pixel shuffle reduces the number of parameters in the model and speeds up the inference of the model, in addition to avoiding the checkerboard effect caused by transposed convolution. As shown in Figs. S7j-m, the 3×3 convolution kernel with a step size of 1 and the dilated convolution kernel with different receptive fields form the branches of the receptive field block (in Fig. S7j, conv2d(3,1,1) represents the 3×3 convolution kernel with a step size of 1 and dilatation rate 1). Compared with the normal convolution, the dilated convolution introduces a hyperparameter called dilatation rate, which is used to define the distance between adjacent elements of the convolution. The larger the dilatation rate is, the larger the receptive field of the dilated convolution is. The sizes of the receptive fields of the receptive field block branches I-IV are calculated to be 3, 7, 9, and 13, respectively. The different scales of the receptive fields help to fully extract the details in the input focus stack and generate high-fidelity holograms.

**S4.2: Description of the band-limited angular spectrum method**

In the FS-Net, the back propagation of the amplitude field and phase field *U*_H_ (*x*, *y*) of the hologram can be expressed as follows:

 (S15)

where ||∙|| means taking the modulus of the expression, *I*_i_ (*x*, *y*) represents the intensity of the reconstructed image of the *i* th layer, F{ } and F^-1^{ } represent the Fourier transform and inverse Fourier transform, respectively. *P*_Z_(*x*, *y*) represents the Zernike phase, *λ*_n_ represents the wavelength of the laser, *z*_0_ represents the initial recording distance, ∆*z* represents the layer spacing of the real 3D scene, *f*_x_ and *f*_y_ represent the horizontal and vertical coordinates of the frequency domain, respectively. *w*(*f*_x_, *f*_y_) is a frequency domain filter for frequency domain constraints and is expressed as follows:

$w(f_{\text{x}},f_{\text{y}})=f_{\text{x}}<[(\frac{2z\cdot\lambda_{\text{n}}}{S\cdot p})^{2}+\lambda^{2}]^{-\frac{1}{2}}\text{ and }f_{\text{y}}<[(\frac{2z\cdot\lambda_{\text{n}}}{V\cdot p})^{2}+\lambda^{2}]^{-\frac{1}{2}}$ (S16)

where *z* represents the diffraction distance, *p* represents the pixel pitch of the spatial light modulator (SLM), *S* and *V* represent the horizontal and vertical resolution of the SLM, respectively.

**S4.3: Description of the loss functions of the FS-Net**

In the image generation task of the deep learning, the mean square error (MSE) loss is commonly used for the approximation of the generated image to the target image. However, the MSE loss often causes smoothing of the generated image. In the training of the FS-Net, in order to learn the focusing features and blurring features of the real 3D scene, a composite loss function consisting of the perceptual (PE) loss, the multiscale structural similarity (MS-SSIM) loss, the total variance (TV) loss, the MSE loss, and the mean absolute error (MAE) loss is used^3^.

The PE loss often uses a pre-trained convolutional neural network (e.g., VGG19) to extract the low-level feature (e.g., texture) and high-level feature (e.g., edges) information of the input target image and to complete the calculation of the pixel difference values in the feature space. The expression of the PE loss is as follows:

$Loss_{\mathrm{PE}}=\sum_{\text{k}} \frac{1}{C_{\mathrm{k}}\times H_{\mathrm{k}}\times W_{\mathrm{k}}}\left\|\text{Ψ}_{\mathrm{k}}(\text{Θ})-\text{Ψ}_{\mathrm{k}}(\hat{\text{Θ}})\right\|_{2}^{2}$ (S17)

where ||∙||2 2 represents to calculate the square of the L2 norm of the expression within the symbol, *Ψ*_k_ represents the *k-*th convolutional layer used for feature extraction in the VGG19, the number *k* is ‘2, 4, 6, 10, 14’, *Θ* represents the intensity of the reconstructed all-in-focus image, $\hat{\text{Θ}}$ represents the intensity of the all-in-focus real 3D scene, *C*_k_×*H*_k_×*W*_k_ represents the channel×height×width of the *k-*th convolutional layer.

In the FS-Net, the MS-SSIM loss is used to measure the structural similarity between the reconstructed defocus image and the target defocus image. The formula of the MS-SSIM loss is expressed as follows:

$Loss_{\mathrm{MS}}=1-\prod_{d=1}^{D} (\frac{2\mu_{\text{g}}\mu_{\text{t}}+c_{1}}{\mu_{\text{g}}^{2}+\mu_{\text{t}}^{2}+c_{1}})^{\beta_{\text{d}}}(\frac{2\sigma_{\text{gt}}+c_{2}}{\sigma_{\text{g}}^{2}+\sigma_{\text{t}}^{2}+c_{2}})^{\gamma_{\text{d}}}$ (S18)

where *d* is a scaling factor, and the height and width of the input image are scaled by a factor of 2^d-1^ (*d*=1, ..., *D*), *μ*_g_ and *μ*_t_ represent the mean values of the reconstructed defocus image and the target defocus image, respectively, *σ*_g_ and *σ*_t_ represent the standard deviations of the reconstructed defocus image and the target defocus image, respectively, *σ*_gt_ represents the covariance of the reconstructed defocus image and the target defocus image, *c*_1_ and *c*_2_ are two constant terms that are used to prevent the denominator from being zero, *β*_d_ and *γ*_d_ stand for the relative importance of the mean and variance terms, respectively.

The TV loss makes the reconstructed all-in-focus image closer to the target all-in-focus image. The basic idea of the TV loss is to utilize the total variance of the image as a measure of the smoothness of the image, thus suppressing noise and artifacts. In the FS-Net, the TV loss is used to constrain the reconstructed all-in-focus image:

$Loss_{\text{TV}}=\sum_{u,v} \sqrt{(p_{\text{u,v-1}}-p_{\text{u,v}})^{2}+(p_{\text{u+1,v}}-p_{\text{u,v}})^{2}}$ (S19)

where *p*_u,v_ represents a pixel point of the input image. The TV loss calculates the square root of the sum of the squares of the differences between each pixel point *p*_u,v_ and the neighboring pixels *p*_u,v-1_ in the horizontal direction and *p*_u+1,v_ in the vertical direction, respectively. After the calculation is complete, the TV loss is obtained by summing the loss values at all pixel points.

In addition, the loss function consisting of the MSE loss and MAE loss is also used for the training of the FS-Net. For the reconstructed all-in-focus image and the reconstructed defocus image, both the MSE loss and the MAE loss of them with respect to the target all-in-focus image and the target defocus image are calculated. The expression of the corresponding loss function is shown as follows:

$\begin{matrix} Loss_{\text{MSE-MAE}}=\frac{1}{l}[(Loss_{\text{MSE}}(I_{\text{rf}},I_{\text{tf}})+0.1\times Loss_{\text{MAE}}(I_{\text{rf}},I_{\text{tf}}) \\ \begin{matrix} \begin{matrix} & \end{matrix} & & \end{matrix}+Loss_{\text{MSE}}(I_{\text{rdf}},I_{\text{tdf}})+0.1\times Loss_{\text{MAE}}(I_{\text{rdf}},I_{\text{tdf}})] \end{matrix}$ (S20)

where *l* represents the number of layers, *I*_rf_ represents the reconstructed all-in-focus image, *I*_tf_ represents the target all-in-focus image, *I*_rdf_ represents the reconstructed defocus image, and *I*_tdf_ represents the target defocus image. In summary, the total loss function of the FS-Net is expressed as follows:

$Loss=\alpha Loss_{\mathrm{PE}}(I_{\text{rf}},I_{\text{tf}})+\beta Loss{}_{\text{MS}}(I_{\text{rdf}},I_{\text{tdf}})+\eta Loss_{\text{TV}}(I_{\text{rf}})+\varepsilon Loss_{\text{MSE-MAE}}$ (S21)

where *α*, *β*, *η*, and *ε* are the coefficients of the PE loss, the MS-SSIM loss, the TV loss, and the MSE-MAE loss, respectively. In the training of the FS-Net, *α*, *β*, *η,* and *ε* are set to be 0.03, 1, 1×10^-6^, and 1 respectively.

**S5: Details of the liquid camera**

**S5.1 Methods for testing the response time and focal power of the liquid camera**

The filled liquid in the proposed liquid lens is a mixture of propylene glycol and tetrabutylammonium chloride with a viscosity of ~56 mPa·s and a refractive index of ~1.43. The response time measurement system consists of a He-Ne laser, a silicon amplification detector, and an oscilloscope. The silicon amplification detector is used to convert the optical signal into a voltage signal. The oscilloscope is used to display the voltage signal. A square wave alternating current with an amplitude of 50 mA and a frequency of 0.25 Hz is used to measure the response time of the liquid camera. After normalizing the voltage signal displayed on the oscilloscope, the time taken from 10% to 90% of the maximum voltage is recorded, which is the response time of the liquid camera.

The focal power of the liquid camera is tested by using an focal power measuring device and applying a current of -50 mA to 60 mA. At an applied current of -50 mA, the focal power of the liquid camera is 34.16 m^-1^. At an applied current of 0 mA, the focal power of the liquid camera is 6.78 m^-1^. At an applied current of 60 mA, the focal power of the liquid camera is -12.09 m^-1^.

**S5.2: Imaging performance testing of the liquid camera**

In order to verify the imaging performance of the liquid camera, the zoom testing is conducted. The elastic membrane liquid lens is placed on a carrier and a mobile phone camera is utilized to capture the entire process of changing the size of the letters. The drive current of the elastic membrane liquid lens is varied from -50 mA to 50 mA, and changing the size of the letters is recorded at different drive current. As shown in Fig. S8, when the drive currents are -50 mA, -20 mA, 20 mA, and 50 mA, the letters captured by the liquid camera change from large to small, and the clarity of the letters remains unchanged, which demonstrates that the fabricated liquid camera has excellent zoom capability.

**
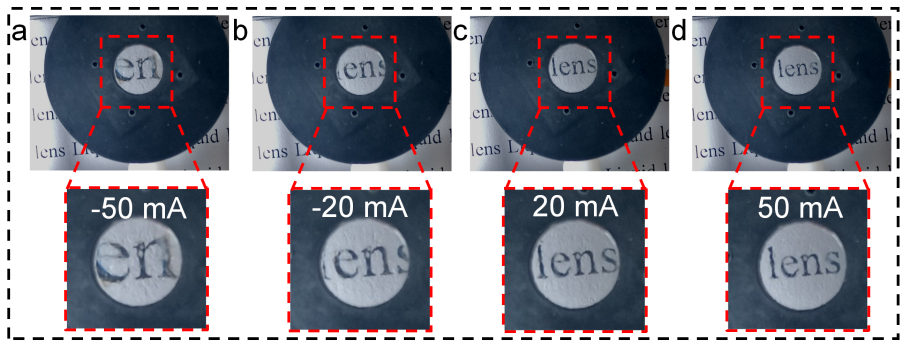
**

**Fig. S8 Zooming process of the liquid camera. a-d** Changes in the letters ‘lens’ when the drive current is changed from -50 mA to 50 mA.

**S6: Additional description of the experiment**

**S6.1: Evaluation metrics for the quality of the holographic reconstructed image**

The PSNR and structural similarity (SSIM) are used to evaluate the quality of the holographic reconstructed image^3^. The PSNR can be expressed as follows:

$\text{PSNR}=20\cdot\lg\left(\frac{1}{\sqrt{\frac{1}{mn}\sum_{i=0}^{m-1} \sum_{j=0}^{n-1} \left\|X(p,q)-Y(p,q)\right\|^{2}}}\right)$ (S22)

where *X*(*p*, *q*) and *Y*(*p*, *q*) represent the target image and the comparison image with a size of *m*×*n*, respectively. The pixel values of the images are taken in the range of 0 to 1. The SSIM can be expressed as follows:

$\text{SSIM}=(\frac{2\mu_{\text{x}}\mu_{\text{y}}+c_{1}}{\mu_{\text{x}}^{2}+\mu_{\text{y}}^{2}+c_{1}})(\frac{2\sigma_{\text{xy}}+c_{2}}{\sigma_{\text{x}}^{2}+\sigma_{\text{y}}^{2}+c_{2}})$ (S23)

where *μ*_x_ and *μ*_y_ represent the mean values of the comparison image and the target image, respectively. *σ*_x_ and *σ*_y_ represent the standard deviation of the comparison image and the target image, respectively. *σ*_xy_ represents the covariance of the comparison image and the target image. *c*_1_ and *c*_2_ are two constant terms that are used to prevent the denominator from being zero.

**S6.2: Loss curves for training and validation processes of the FS-Net**

The training loss curve and validation loss curve of the FS-Net are shown in Fig. S9. As shown in Fig. S9a, at the end of the training stage, the training loss value of the red channel is 0.022, the training loss value of the green channel is 0.024, and the training loss value of the blue channel is 0.026. As shown in Fig. S9b, at the end of the validation stage, the validation loss value of the red channel is 0.042, the validation loss value of the green channel is 0.034, and the validation loss value of the blue channel is 0.030.


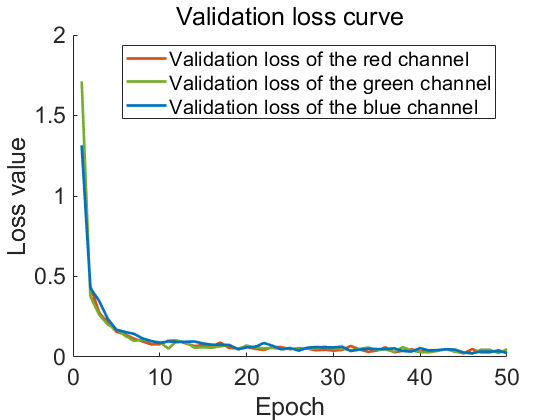

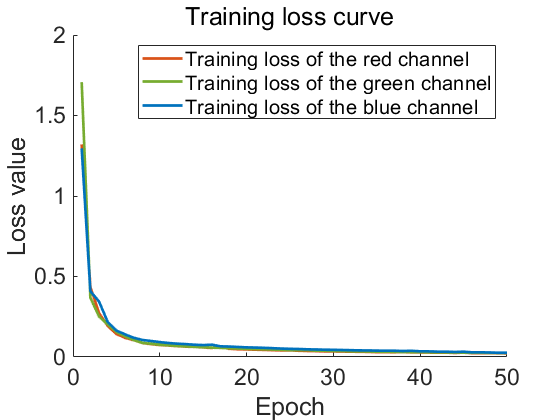


**a**

**b**

**Fig. S9 Loss curves of the FS-Net. a** Training loss curve. **b** Validation loss curve.

**References**

1. Gur, S. & Wolf, L. Single image depth estimation trained via depth from defocus cues. In *2019 IEEE/CVF Conference on Computer Vision and Pattern Recognition (CVPR)* 7675–7684 (IEEE, Long Beach, 2019).
2. Maimone, A. *et al.* Holographic near-eye displays for virtual and augmented reality. *ACM Transactions on Graphics (TOG)* **36**, 85 (2017).
3. Wang, D. *et al.* Liquid lens based holographic camera for real 3D scene hologram acquisition using end-to-end physical model-driven network. *Light: Science & Applications* **13**, 62 (2024).
